# Predictive value of non-high-density lipoprotein cholesterol and remnant cholesterol for progression of non-culprit coronary lesions with QFR ≤ 0.80: A retrospective cohort study

**DOI:** 10.1097/MD.0000000000050907

**Published:** 2026-10-02

**Authors:** Guoran Ruan, Qianqian Guan, Jiaqing Li, Guo Li, Haojin Ren

**Affiliations:** a Department of Cardiology, Puren Hospital Affiliated to Wuhan University of Science and Technology, Wuhan City, Hubei Province, China.

**Keywords:** lesion progression, non-culprit lesion, non-high-density lipoprotein cholesterol, quantitative flow ratio, remnant cholesterol

## Abstract

To investigate the predictive value of non-high-density lipoprotein cholesterol (non-HDL-C) and remnant cholesterol (remnant-C) for the progression of non-culprit coronary lesions with quantitative flow ratio ≤ 0.80. A total of 214 patients with coronary artery disease undergoing percutaneous coronary intervention from January 2022 to March 2024 were consecutively enrolled, with 321 non-culprit lesions. All patients completed at least 24 months of clinical follow-up. Based on follow-up quantitative coronary angiography assessment, patients were divided into lesion progression and non-progression groups. Baseline clinical data, laboratory parameters, and coronary angiography characteristics were collected. Univariate and multivariate logistic regression analyses were performed to assess the independent predictive value of non-HDL-C and remnant-C for lesion progression. Predictive performance was compared using receiver operating characteristic curves, and subgroup analyses were conducted. During follow-up, 62 patients (29.0%) experienced non-culprit lesion progression. The median angiographic follow-up interval was 14.2 months (interquartile range, 10.8–18.1; range, 6.1–23.8) overall and did not differ significantly between the progression and non-progression groups (14.6 vs 14.0 months, *P* = .47). Multivariable logistic regression showed that, after adjustment for prespecified clinical covariates, both non-HDL-C (odds ratio = 1.68, 95% confidence interval: 1.22–2.31, *P* = .001) and remnant-C (odds ratio = 1.52, 95% confidence interval: 1.24–1.86, *P* < .001) were independently associated with lesion progression. The apparent areas under the curves were 0.72 for non-HDL-C and 0.70 for remnant-C; after adding clinical variables, the apparent areas under the curves were 0.84 and 0.83, respectively. Numerically larger effect estimates were observed in patients with diabetes mellitus, multivessel disease, and renal dysfunction, but all interaction *P* values were >.05. Non-HDL-C and remnant-C are independent predictors of progression of non-culprit coronary lesions with quantitative flow ratio ≤ 0.80. Combining them with traditional risk factors significantly improves predictive performance, offering clinical value for high-risk patient identification and secondary prevention optimization.

## 1. Introduction

Percutaneous coronary intervention (PCI) is the cornerstone of revascularization in patients with coronary artery disease. However, despite successful treatment of culprit lesions, residual non-culprit lesions remain an important source of future major adverse cardiovascular events.^[1]^ The prospective study showed that approximately 12% of patients with acute coronary syndrome (ACS) experience major adverse cardiovascular events due to non-culprit lesion progression during 3-year follow-up.^[2]^ Recent imaging studies further indicate that non-culprit lesion progression, rather than in-stent restenosis or in-stent thrombosis, is the predominant cause of long-term adverse events after PCI.^[3]^ Therefore, identifying high-risk non-culprit lesions and determining the factors driving their progression have important clinical significance.

Quantitative flow ratio (QFR) is an emerging angiography-based, wire-free technique for functional assessment of coronary stenosis, derived from fractional flow reserve.^[4]^ Previous studies have shown that functionally significant lesions with QFR ≤ 0.80 are closely associated with myocardial ischemia and adverse clinical outcomes.^[5]^ Nevertheless, even among functionally significant non-culprit lesions, there is substantial individual variability in anatomical progression; some lesions remain stable over long periods, whereas others progress rapidly and even lead to acute coronary events. Therefore, further identifying high-risk populations among lesions with QFR ≤ 0.80 is of great value for optimizing follow-up strategies and secondary prevention.

Dyslipidemia is a core driver of atherosclerosis progression. Traditionally, low-density lipoprotein cholesterol (LDL-C) has been the primary target of lipid-lowering therapy. However, a considerable proportion of patients still experience residual cardiovascular risk even after achieving LDL-C targets.^[6]^ In recent years, non-high-density lipoprotein cholesterol (non-HDL-C) and remnant cholesterol (remnant-C) have gained substantial attention as emerging lipid parameters. Non-HDL-C reflects the cholesterol burden of all atherogenic lipoproteins, including low-density lipoprotein (LDL), very low-density lipoprotein, intermediate-density lipoprotein, and lipoprotein(a).^[7]^ Remnant cholesterol represents the cholesterol content of triglyceride-rich lipoproteins and their remnants and has been demonstrated to be closely associated with atherosclerosis progression and residual inflammatory risk.^[8]^ Previous studies have shown that non-HDL-C and remnant-C may have superior predictive value for cardiovascular events compared with LDL-C; however, their predictive value for the progression of non-culprit lesions with QFR ≤ 0.80 remains unclear.

Therefore, this retrospective cohort study aims to investigate the predictive value of non-HDL-C and remnant-C for the progression of non-culprit coronary lesions with QFR ≤ 0.80, providing evidence-based support for risk stratification and individualized lipid-lowering therapy in patients after PCI.

## 2. Methods

### 2.1. Study design

This study was approved by the Ethics Committee of Puren Hospital Affiliated to Wuhan University of Science and Technology. This was a single-center retrospective cohort study. Consecutive patients with coronary artery disease who underwent coronary angiography and PCI in our hospital from January 2022 to March 2024 were enrolled. Clinical follow-up continued for at least 24 months for all patients, with the clinical follow-up cutoff in March 2026. In contrast, the angiographic follow-up interval used for assessment of lesion progression was the time from the index coronary angiography to the follow-up coronary angiography and, according to the inclusion criteria, ranged from 6 to 24 months. The median angiographic follow-up interval was 14.2 months (interquartile range [IQR], 10.8–18.1; range, 6.1–23.8). A total of 214 eligible patients with 321 non-culprit lesions were finally included. The institutional ethics committee waived informed consent because of the retrospective observational design.

### 2.2. Inclusion and exclusion criteria

Inclusion criteria were as follows: age ≥18 years; undergoing coronary angiography and stent implantation in at least 1 major coronary artery (left anterior descending, left circumflex, and right coronary artery) for stable angina or non-ST-segment elevation ACS; presence of at least 1 non-culprit coronary lesion (diameter stenosis ≥30% and <100%) with QFR ≤ 0.80; undergoing planned or clinically driven follow-up coronary angiography within 6 to 24 months after the procedure; available imaging data for QFR and quantitative coronary angiography (QCA) analysis at baseline and follow-up; and complete baseline lipid data (at least including total cholesterol [TC], triglycerides, high-density lipoprotein cholesterol [HDL-C], and LDL-C).

Exclusion criteria were as follows: previous coronary artery bypass grafting; severe valvular heart disease, cardiomyopathy, or heart failure (LVEF < 40%); target lesion revascularization or in-stent restenosis affecting non-culprit lesion assessment at baseline or during follow-up; occurrence of myocardial infarction during follow-up (which may accelerate diffuse lesion progression); poor baseline or follow-up imaging quality precluding QFR/QCA analysis; and severe hepatic or renal dysfunction or poor adherence to statin therapy.

### 2.3. Sample size and model stability

This study was a retrospective exploratory cohort study, and no a priori sample size calculation was performed. A total of 214 patients were included in the patient-level analysis, of whom 62 (29.0%) experienced progression of at least 1 non-culprit lesion during angiographic follow-up. Because the final multivariable logistic regression analyses were conducted at the patient level, model complexity was considered in relation to these 62 patient-level outcome events rather than the number of lesion-level events. Each final model included 1 lipid exposure variable (non-HDL-C or remnant-C) and 7 clinical covariates (age, sex, diabetes mellitus, systolic blood pressure ≥140 mm Hg, multivessel disease, systemic immune-inflammation index [SII], and estimated glomerular filtration rate [eGFR]), corresponding to approximately 8 regression parameters and an events-per-variable ratio of approximately 7.8. Given the relatively limited number of outcome events, the multivariable models may be susceptible to overfitting and imprecision in regression coefficient estimates. Therefore, the regression analyses should be considered exploratory, and the estimated associations require confirmation in larger independent cohorts.

### 2.4. Definition of lipid exposure factors

The core exposure variables of this study were baseline non-HDL-C and remnant-C.^[9,10]^ All lipid parameters were obtained from fasting venous blood samples collected during hospitalization before the first coronary angiography and QFR assessment. Non-HDL-C was calculated as follows: total cholesterol (TC) − high-density lipoprotein cholesterol (HDL-C), that is,


non−HDL−C(mmol/L)=TC−HDL−C.

Remnant cholesterol was estimated using the widely accepted clinical formula:


$$\begin{array}{c} Remnant\;cholesterol\;\left(mmol/L\right)=TC-HDL-C-low-density\;lipoprotein\;cholesterol\;\left(LDL-C\right). \end{array}$$

### 2.5. Outcome definition

The outcome measure of this study was defined based on the comparison between follow-up QCA and baseline results. Imaging analysis of all non-culprit lesions was independently completed by 2 interventional cardiologists blinded to clinical and lipid data, with the same angiographic projection selected to ensure comparability.

#### 2.5.1. Primary outcome

The primary outcome was anatomical progression of non-culprit lesions, defined as meeting any of the following QCA criteria: absolute increase in diameter stenosis percentage (%DS) ≥ 10%; absolute decrease in lumen diameter of the lesion segment ≥0.4 mm; progression from baseline stenosis <50% to ≥50%, or from baseline stenosis ≥50% to ≥70%; and progression to total occlusion (thrombolysis in myocardial infarction flow grade 0–1). The primary outcome was assessed using 2 analytical levels: lesion-based analysis (each eligible non-culprit lesion independently included) and patient-based analysis (a patient considered to have an outcome event if at least 1 non-culprit lesion met the above progression criteria), allowing comprehensive evaluation of the impact of lipid parameters at different analytical levels.

#### 2.5.2. Secondary outcomes

Secondary outcomes included the following 2 categories: rapid lesion progression, defined as an absolute increase in diameter stenosis percentage of ≥20% or direct progression to total occlusion; and non-culprit vessel revascularization driven by lesion progression, defined as PCI or coronary artery bypass grafting performed because of ischemia-related symptoms or objective evidence of ischemia attributable to progression of the non-culprit lesion.

Because all eligible non-culprit lesions in the present study were required to have a baseline QFR ≤0.80, lesions with baseline QFR >0.80 were not included. Accordingly, transition from a baseline QFR >0.80 to a follow-up QFR ≤0.80 was not evaluated as a secondary outcome.

### 2.6. Data collection

Baseline patient data were collected through the electronic medical record system, including the following main contents: demographic characteristics, including age, sex, body mass index, and obesity (body mass index ≥ 28 kg/m^2^); cardiovascular risk factors, including hypertension (defined as systolic blood pressure ≥140 mm Hg and/or diastolic blood pressure ≥90 mm Hg, or a documented history of hypertension), diabetes mellitus (defined as fasting blood glucose ≥7.0 mmol/L, HbA1c ≥ 6.5%, or a documented history of diabetes), smoking history (current or former smoking), pack-years of smoking, and prior myocardial infarction; clinical diagnosis and lesion burden, including type of clinical diagnosis (stable angina or non-ST-segment elevation ACS), multivessel disease (≥50% stenosis in ≥2 major coronary arteries), and number of non-culprit lesions; laboratory parameters, including TC, triglycerides, HDL-C, LDL-C, non-HDL-C, remnant-C, lipoprotein(a), fasting blood glucose, glycated hemoglobin (HbA1c), high-sensitivity C-reactive protein, and fibrinogen; systemic immune-inflammation index (SII) = platelet count × neutrophil count/lymphocyte count; renal function parameters, including serum creatinine and eGFR calculated using the Chronic Kidney Disease Epidemiology Collaboration formula; medications, including use of statins (distinguishing moderate-intensity and high-intensity), ezetimibe, proprotein convertase subtilisin/kexin type 9 inhibitors, angiotensin-converting enzyme inhibitor/angiotensin II receptor blocker, and beta-blockers at baseline and during follow-up; and coronary angiography and functional parameters, including lesion characteristics (target vessel location, bifurcation lesion, long lesion [≥20 mm], calcified lesion, diameter stenosis percentage, minimal lumen diameter, and reference vessel diameter) obtained by QCA and functional severity of lesions assessed by QFR.

### 2.7. Statistical analysis

Data analysis was performed using IBM SPSS Statistics, version 26.0 (IBM Corp.) and R, version 4.2 (R Foundation for Statistical Computing). Normality of continuous variables was assessed using the Kolmogorov–Smirnov test. Normally distributed variables were expressed as mean ± standard deviation, with comparisons between groups using the independent-samples *t* test. Non-normally distributed variables were expressed as median (IQR), with comparisons between groups using the Mann–Whitney *U* test. Categorical variables were expressed as frequency (percentage), with comparisons between groups using the χ^2^ test or Fisher’s exact test. The angiographic follow-up interval (time between baseline and follow-up coronary angiography) was summarized as median (IQR) and range and was compared between the progression and non-progression groups using the Mann–Whitney *U* test.

Univariate logistic regression was performed using patient-level (n = 214) and lesion-level (n = 321) analysis units to identify factors associated with non-culprit lesion progression. Because subsequent multivariable analyses focused on patient-level risk factors, model stability was evaluated according to the 62 patient-level progression events. Variables with *P* < .10 in univariate analysis and clinically relevant covariates were considered for multivariable modeling. Given the high collinearity between non-HDL-C and remnant-C (Spearman correlation coefficient = 0.71), 2 separate multivariable models were constructed: Model A with non-HDL-C as the core exposure and Model B with remnant-C as the core exposure. Both models were adjusted for age, sex, diabetes mellitus, systolic blood pressure ≥140 mm Hg, multivessel disease, SII, and eGFR. Receiver operating characteristic (ROC) curves were used to describe the apparent discriminatory ability of each predictor and model within the same derivation cohort, with comparison of the area under the curve (AUC) using the DeLong test. No internal resampling or external validation was performed; therefore, AUC estimates were considered potentially optimistic. Subgroup analyses were stratified by diabetes mellitus, hypertension, sex, age, statin intensity, multivessel disease, systolic blood pressure, and eGFR. Interaction *P* values were calculated using the likelihood ratio test, and subgroup differences were interpreted only when the interaction test was statistically significant. A two-sided *P* < .05 was considered statistically significant. Because only 62 patient-level progression events were observed, the number of covariates in the multivariable models was kept limited, and the resulting estimates were interpreted cautiously given the potential for overfitting.

## 3. Results

### 3.1. Baseline characteristics and comparison between groups

Based on whether anatomical progression of non-culprit lesions occurred during follow-up, patients were divided into a progression group (62 cases, 29.0%) and a non-progression group (152 cases, 71.0%). Baseline characteristics of the 2 groups are shown in Table 1. The median interval between baseline and follow-up coronary angiography was 14.2 months (IQR, 10.8–18.1; range, 6.1–23.8) in the overall cohort. The interval was 14.6 months (IQR, 11.1–18.4) in the progression group and 14.0 months (IQR, 10.6–17.9) in the non-progression group, with no statistically significant between-group difference (*P* = .47). The proportion of patients with diabetes mellitus (51.6% vs 30.3%, *P* = .004) and the number of non-culprit lesions (2.0 vs 1.0, *P* = .036) were significantly higher in the progression group than in the non-progression group. Regarding laboratory parameters, levels of non-HDL-C, remnant-C, lipoprotein(a), fasting blood glucose, HbA1c, and SII were significantly higher, while HDL-C and eGFR levels were significantly lower in the progression group compared with the non-progression group (all *P* < .05). No statistically significant differences were observed between the 2 groups in age, sex, hypertension, smoking history, or medication use (all *P* > .05).

**Table 1:** Comparison of baseline characteristics according to lesion progression at the patient level (patient-level analysis).

Variable	Progression group (N = 62)	Non-progression group (N = 152)	*P* value
Demographic characteristics			
Age (yr), mean ± SD	63.5 ± 9.6	61.8 ± 10.6	.258
Male, n (%)	44 (71.0)	104 (68.4)	.713
BMI (kg/m^2^), mean ± SD	25.8 ± 3.2	25.5 ± 3.0	.512
BMI ≥ 28 kg/m^2^ (obesity), n (%)	18 (29.0)	32 (21.1)	.214
Cardiovascular risk factors			
Hypertension, n (%)	42 (67.7)	94 (61.8)	.421
Systolic blood pressure ≥140 mm Hg, n (%)	28 (45.2)	52 (34.2)	.132
Diabetes mellitus, n (%)	32 (51.6)	46 (30.3)	.004
Smoking history (current or former), n (%)	34 (54.8)	68 (44.7)	.179
Pack-years of smoking, median (IQR)	32.5 (20.0–48.0)	26.0 (15.0–40.0)	.082
Prior myocardial infarction, n (%)	14 (22.6)	28 (18.4)	.482
Clinical diagnosis and lesion burden			
NSTE-ACS (vs stable angina), n (%)	38 (61.3)	84 (55.3)	.424
Multivessel disease (≥2 vessels), n (%)	46 (74.2)	92 (60.5)	.058
Number of non-culprit lesions, median (IQR)	2.0 (1.0–2.0)	1.0 (1.0–2.0)	.036
Laboratory parameters			
Total cholesterol (mmol/L), mean ± SD	4.92 ± 1.12	4.38 ± 1.04	.001
Triglycerides (mmol/L), median (IQR)	1.72 (1.28–2.46)	1.48 (1.06–2.14)	.048
HDL-C (mmol/L), mean ± SD	1.00 ± 0.24	1.08 ± 0.26	.032
LDL-C (mmol/L), mean ± SD	2.82 ± 0.90	2.62 ± 0.82	.118
Non-HDL-C (mmol/L), mean ± SD	3.86 ± 0.96	3.32 ± 0.92	<.001
Remnant cholesterol (mmol/L), median (IQR)	0.98 (0.74–1.26)	0.72 (0.54–0.96)	<.001
Lipoprotein(a) (mg/L), median (IQR)	185 (112–286)	142 (86–224)	.028
Glucose metabolism parameters			
Fasting blood glucose (mmol/L), mean ± SD	6.48 ± 1.82	5.86 ± 1.46	.008
HbA1c (%), mean ± SD	6.62 ± 0.96	6.12 ± 0.84	<.001
Inflammatory and immune parameters			
hs-CRP (mg/L), median (IQR)	2.48 (1.08–4.96)	1.96 (0.88–4.08)	.094
Systemic immune-inflammation index (SII, ×10^9^/L), median (IQR)	568 (412–786)	486 (358–652)	.022
Fibrinogen (g/L), mean ± SD	3.42 ± 0.86	3.18 ± 0.74	.056
Renal function parameters			
eGFR (mL/min/1.73 m^2^), mean ± SD	78.6 ± 18.2	84.2 ± 17.8	.038
Serum creatinine (μmol/L), mean ± SD	82.5 ± 18.6	78.2 ± 16.4	.112
Medication, n (%)			
High-intensity statin	24 (38.7)	62 (40.8)	.778
Ezetimibe	12 (19.4)	34 (22.4)	.630
PCSK9 inhibitor	4 (6.5)	12 (7.9)	.712
ACEI/ARB	38 (61.3)	90 (59.2)	.778
Beta-blocker	42 (67.7)	102 (67.1)	.930

ACEI = angiotensin-converting enzyme inhibitor, ACS = acute coronary syndrome, ARB = angiotensin II receptor blocker, BMI = body mass index, eGFR = estimated glomerular filtration rate, HbA1c = glycated hemoglobin, HDL-C = high-density lipoprotein cholesterol, hs-CRP = high-sensitivity C-reactive protein, IQR = interquartile range, LDL-C = low-density lipoprotein cholesterol, NSTE-ACS = non-ST-segment elevation acute coronary syndrome, PCSK9 = proprotein convertase subtilisin/kexin type 9, SD = standard deviation.

### 3.2. Comparison of lesion characteristics between groups

To further explore the impact of lesion-specific features on progression, comparisons were performed at the lesion level (321 non-culprit lesions in total), as shown in Table 2. No statistically significant differences were observed between the progression group (78 lesions, 24.3%) and the non-progression group (243 lesions, 75.7%) in terms of target vessel distribution, bifurcation lesions, long lesions, calcified lesions, baseline diameter stenosis percentage, minimal lumen diameter, reference vessel diameter, or QFR value (all *P* > .05).

**Table 2:** Comparison of lesion characteristics according to lesion progression (lesion-level analysis).

Variable	Progression group (N = 78 lesions)	Non-progression group (N = 243 lesions)	*P* value
Lesion characteristics			
Target vessel: left anterior descending (LAD), n (%)	42 (53.8)	118 (48.6)	.420
Bifurcation lesion, n (%)	28 (35.9)	68 (28.0)	.172
Long lesion (≥20 mm), n (%)	32 (41.0)	78 (32.1)	.142
Calcified lesion, n (%)	22 (28.2)	56 (23.0)	.350
Baseline diameter stenosis (%), mean ± SD	58.6 ± 12.4	56.2 ± 11.8	.135
Baseline minimal lumen diameter (mm), mean ± SD	1.28 ± 0.42	1.34 ± 0.38	.263
Reference vessel diameter (mm), mean ± SD	2.86 ± 0.52	2.92 ± 0.48	.354
Baseline quantitative flow ratio (QFR), mean ± SD	0.72 ± 0.08	0.74 ± 0.07	.082

SD = standard deviation.

### 3.3. Univariate logistic regression analysis

To screen for potential risk factors for non-culprit lesion progression, univariate logistic regression analysis was performed at both the patient level (214 cases) and the lesion level (321 lesions). Results are shown in Tables 3 and 4.

**Table 3:** Univariate logistic regression analysis of factors associated with non-culprit lesion progression (patient-level, N = 214).

Variable	OR (95% CI)	*P* value
Lipid parameters		
Non-HDL-C (per 1 mmol/L increase)	1.78 (1.32–2.40)	<.001
Remnant cholesterol (per 0.1 mmol/L increase)	1.48 (1.22–1.80)	<.001
LDL-C (per 1 mmol/L increase)	1.32 (0.88–1.98)	.182
Triglycerides (per 1 mmol/L increase)	1.26 (0.96–1.65)	.096
HDL-C (per 0.2 mmol/L increase)	0.80 (0.64–1.00)	.048
Lipoprotein(a) (per 50 mg/L increase)	1.20 (0.98–1.47)	.076
Inflammatory and immune parameters		
hs-CRP (per 1 mg/L increase)	1.09 (0.96–1.24)	.186
Systemic immune-inflammation index (SII; per 100 × 10^9^/L increase)	1.11 (1.01–1.22)	.028
Fibrinogen (per 1 g/L increase)	1.38 (0.94–2.03)	.102
Glucose metabolism parameters		
Fasting blood glucose (per 1 mmol/L increase)	1.18 (1.04–1.34)	.010
HbA1c (per 1% increase)	1.44 (1.12–1.85)	.004
Diabetes mellitus (vs no)	2.24 (1.22–4.11)	.009
Demographic characteristics		
Age (per 10-yr increase)	1.14 (0.86–1.51)	.364
Male (vs female)	1.28 (0.68–2.41)	.442
BMI (≥28 kg/m^2^ vs <24)	1.58 (0.94–2.66)	.084
Cardiovascular risk factors		
Hypertension (vs no)	1.24 (0.68–2.26)	.482
Systolic blood pressure ≥140 mm Hg (vs <120)	1.48 (0.96–2.28)	.076
Smoking history (vs no)	1.28 (0.68–2.41)	.442
Pack-years of smoking (per 10 pack-year increase)	1.20 (0.98–1.47)	.078
Clinical diagnosis and lesion burden		
NSTE-ACS (vs stable angina)	1.26 (0.70–2.27)	.442
Multivessel disease (≥2 vessels vs single vessel)	1.86 (1.10–3.14)	.020
Total number of non-culprit lesions (per 1 lesion increase)	1.32 (1.06–1.64)	.012
Prior myocardial infarction (vs no)	1.52 (0.86–2.68)	.148
Renal function parameters		
eGFR (per 10 mL/min/1.73 m^2^ decrease)	1.18 (1.01–1.38)	.036
Serum creatinine (per 10 μmol/L increase)	1.10 (0.94–1.29)	.238
Treatment factors		
High-intensity statin (vs moderate-intensity)	0.74 (0.40–1.37)	.338
Ezetimibe (vs no)	0.68 (0.32–1.45)	.318
PCSK9 inhibitor (vs no)	0.54 (0.24–1.21)	.134
ACEI/ARB (vs no)	0.82 (0.46–1.46)	.502
Beta-blocker (vs no)	1.08 (0.60–1.94)	.798

ACEI = angiotensin-converting enzyme inhibitor, ACS = acute coronary syndrome, ARB = angiotensin II receptor blocker, BMI = body mass index, CI = confidence interval, eGFR = estimated glomerular filtration rate, HbA1c = glycated hemoglobin, HDL-C = high-density lipoprotein cholesterol, LDL-C = low-density lipoprotein cholesterol, NSTE-ACS = non-ST-segment elevation acute coronary syndrome, OR = odds ratio, PCSK9 = proprotein convertase subtilisin/kexin type 9.

**Table 4:** Univariate logistic regression analysis of factors associated with non-culprit lesion progression (lesion-level, N = 321).

Variable	OR (95% CI)	*P* value
Lesion characteristics		
Target vessel LAD (vs non-LAD)	1.23 (0.74–2.05)	.425
Baseline diameter stenosis (per 10% increase)	1.18 (0.92–1.52)	.186
Baseline minimal lumen diameter (per 0.1 mm decrease)	1.08 (0.94–1.24)	.286
Reference vessel diameter (per 0.1 mm decrease)	1.04 (0.91–1.19)	.558
Baseline QFR (per 0.1 decrease)	1.34 (0.96–1.87)	.086
Bifurcation lesion (vs non-bifurcation)	1.56 (0.98–2.48)	.062
Long lesion (≥20 mm vs <20 mm)	1.42 (0.94–2.15)	.098
Calcified lesion (vs no)	1.28 (0.84–1.95)	.252

CI = confidence interval, OR = odds ratio, QFR = quantitative flow ratio.

Patient-level analysis (Table 3) showed that non-HDL-C, remnant-C, diabetes mellitus, HbA1c, multivessel disease, total number of non-culprit lesions, SII, and decreased eGFR were significantly associated with the risk of lesion progression (all *P* < .05), whereas elevated HDL-C had a protective effect (*P* = .048).

Lesion-level analysis (Table 4) showed that although certain lesion characteristics such as bifurcation lesions, long lesions, and lower baseline QFR showed certain trends, none reached statistical significance (all *P* > .05), suggesting that the anatomical and functional features of the lesions themselves had limited predictive value for the outcome in this study. Therefore, subsequent multivariate analyses focused on patient-level risk factors.

### 3.4. Multivariate logistic regression analysis

Based on the univariate analysis results, variables significant at the patient level (*P* < .10) were entered into multivariate logistic regression models to evaluate the independent predictive value of non-HDL-C and remnant-C for non-culprit lesion progression. Given the high collinearity between the 2, Model A (with non-HDL-C as the core exposure) and Model B (with remnant-C as the core exposure) were constructed separately, adjusting for age, sex, diabetes mellitus, systolic blood pressure ≥140 mm Hg, multivessel disease, SII, and eGFR. Results are shown in Table 5.

**Table 5:** Multivariate logistic regression analysis of factors associated with non-culprit lesion progression (patient-level analysis, N = 214).

Variable	Model A (with non-HDL-C as core)		Model B (with remnant cholesterol as core)	
	OR (95% CI)	*P* value	OR (95% CI)	*P* value
Non-HDL-C (per 1 mmol/L increase)	1.68 (1.22–2.31)	.001	–	–
Remnant cholesterol (per 0.1 mmol/L increase)	–	–	1.52 (1.24–1.86)	<.001
Diabetes mellitus (vs no)	2.24 (1.22–4.11)	.009	2.32 (1.26–4.27)	.007
Systolic blood pressure ≥140 mm Hg (vs <120)	1.58 (1.06–2.36)	.024	1.62 (1.08–2.43)	.019
Multivessel disease (≥2 vessels vs single vessel)	1.96 (1.18–3.26)	.009	2.02 (1.22–3.35)	.006
SII (per 100 × 10^9^/L increase)	1.14 (1.02–1.27)	.022	1.16 (1.04–1.29)	.008
eGFR (per 10 mL/min/1.73 m^2^ decrease)	1.22 (1.02–1.46)	.028	1.23 (1.03–1.47)	.022
Age (per 10 yr increase)	1.18 (0.90–1.55)	.232	1.14 (0.86–1.51)	.354
Male (vs female)	1.28 (0.70–2.34)	.424	1.34 (0.74–2.43)	.338

CI = confidence interval, eGFR = estimated glomerular filtration rate, non-HDL-C = non-high-density lipoprotein cholesterol, OR = odds ratio, SII = systemic immune-inflammation index.

Model A showed that non-HDL-C was an independent predictor of lesion progression (odds ratio [OR] = 1.68, 95% confidence interval [CI]: 1.22–2.31, *P* = .001). Model B showed that remnant-C was also an independent predictor (OR = 1.52, 95% CI: 1.24–1.86, *P* < .001). In both models, diabetes mellitus, systolic blood pressure ≥140 mm Hg, multivessel disease, elevated SII, and decreased eGFR remained independently significant (all *P* < .05), whereas age and sex did not reach statistical significance.

### 3.5. ROC curve analysis

ROC curve analysis was performed to describe the discrimination of non-HDL-C, remnant-C, and the corresponding multivariable models for patient-level lesion progression. The AUC was 0.72 for non-HDL-C alone and 0.70 for remnant-C alone. After the addition of the selected clinical variables, the apparent AUC increased to 0.84 (95% CI: 0.78–0.90) for Model A and 0.83 (95% CI: 0.77–0.89) for Model B. Both apparent AUCs were higher than those of the corresponding lipid markers alone (DeLong test, both *P* < .001). Because model development and discrimination assessment were performed in the same cohort without internal or external validation, these values should be interpreted as in-sample estimates and may overestimate model performance in new patients (Table 6).

**Table 6:** ROC curve analysis of non-HDL-C and remnant cholesterol for predicting non-culprit lesion progression (patient-level, N = 214).

Predictor/model	AUC (95% CI)	Optimal cutoff	Sensitivity (%)	Specificity (%)	Youden index
Non-HDL-C	0.72 (0.65–0.79)	3.58 mmol/L	68.4	71.2	0.396
Remnant cholesterol	0.70 (0.63–0.77)	0.85 mmol/L	64.1	68.7	0.328
Model A (with non-HDL-C)*	0.84 (0.78–0.90)	–	78.6	82.4	0.610
Model B (with remnant cholesterol)†	0.83 (0.77–0.89)	–	76.8	81.2	0.580

AUC = area under the curve, CI = confidence interval, eGFR = estimated glomerular filtration rate, non-HDL-C = non-high-density lipoprotein cholesterol, ROC = receiver operating characteristic, SII = systemic immune-inflammation index.

* Model A includes non-HDL-C, diabetes mellitus, systolic blood pressure ≥140 mm Hg, multivessel disease, SII, and eGFR.

† Model B includes remnant cholesterol, diabetes mellitus, systolic blood pressure ≥140 mm Hg, multivessel disease, SII, and eGFR.

### 3.6. Subgroup analysis

Subgroup analyses were performed according to diabetes mellitus, hypertension, sex, age, statin intensity, multivessel disease, systolic blood pressure, and eGFR. The results are shown in Table 7.

**Table 7:** Subgroup analysis of the predictive value of non-HDL-C and remnant cholesterol for non-culprit lesion progression (patient-level analysis).

Subgroup category	Number of patients (N = 214)	Model A (non-HDL-C)*		Model B (remnant cholesterol)†		*P* for interaction	
		OR (95% CI)	*P* value	OR (95% CI)	*P* value	Model A	Model B
Diabetes mellitus							
Yes	78	1.82 (1.22–2.72)	.003	1.64 (1.26–2.14)	<.001	.482	.356
No	136	1.58 (1.04–2.40)	.032	1.42 (1.08–1.87)	.014		
Hypertension							
Yes	136	1.72 (1.20–2.46)	.003	1.56 (1.22–2.00)	.001	.684	.572
No	78	1.62 (0.98–2.68)	.06	1.46 (1.02–2.09)	.038		
Sex							
Male	148	1.68 (1.18–2.39)	.004	1.52 (1.20–1.92)	.001	.956	.872
Female	66	1.66 (0.96–2.87)	.068	1.50 (1.00–2.25)	.05		
Age							
≥65 yr	98	1.82 (1.20–2.76)	.005	1.64 (1.22–2.20)	.001	.324	.286
<65 yr	116	1.52 (0.98–2.36)	.062	1.38 (1.00–1.90)	.05		
Statin intensity							
High-intensity	86	1.72 (1.12–2.64)	.013	1.56 (1.16–2.10)	.004	.768	.684
Moderate-intensity	112	1.64 (1.08–2.49)	.02	1.48 (1.10–1.99)	.01		
Multivessel disease							
≥2 vessels	138	1.82 (1.24–2.67)	.002	1.64 (1.26–2.14)	<.001	.286	.242
Single vessel	76	1.46 (0.86–2.48)	.162	1.32 (0.90–1.94)	.152		
Systolic blood pressure							
≥140 mm Hg	80	1.78 (1.14–2.78)	.011	1.60 (1.18–2.17)	.003	.624	.568
<140 mm Hg	134	1.62 (1.06–2.48)	.026	1.46 (1.08–1.97)	.014		
eGFR							
<60 mL/min/1.73 m^2^	42	1.92 (1.12–3.29)	.018	1.72 (1.20–2.46)	.003	.386	.342
≥60 mL/min/1.73 m^2^	172	1.62 (1.14–2.30)	.007	1.46 (1.16–1.84)	.002		

CI = confidence interval, eGFR = estimated glomerular filtration rate, non-HDL-C = non-high-density lipoprotein cholesterol, OR = odds ratio, SII = systemic immune-inflammation index.

* Model A included non-HDL-C, diabetes mellitus, systolic blood pressure ≥140 mm Hg, multivessel disease, SII, and eGFR.

† Model B included remnant cholesterol, diabetes mellitus, systolic blood pressure ≥140 mm Hg, multivessel disease, SII, and eGFR.

The associations of non-HDL-C and remnant-C with lesion progression were generally directionally consistent across the examined subgroups. Numerically higher OR estimates were observed among patients with diabetes mellitus, multivessel disease, and eGFR <60 mL/min/1.73 m^2^ compared with their respective reference subgroups. However, all *P* values for interaction were >.05, indicating no statistically significant evidence of heterogeneity or effect modification across these subgroups. Therefore, these subgroup findings should be interpreted as exploratory.

## 4. Discussion

This retrospective cohort study found that baseline non-HDL-C and remnant-C levels were independent predictors of anatomical progression of non-culprit coronary lesions within 24 months in patients with QFR ≤ 0.80. In the patient-level multivariable analyses, higher non-HDL-C and remnant-C levels remained independently associated with lesion progression after adjustment for selected clinical covariates. However, given the 62 patient-level events and the number of parameters included in the models, these adjusted estimates should be interpreted cautiously and regarded as exploratory.^[10]^ ROC curve analysis indicated that the predictive performance of non-HDL-C or remnant-C alone was moderate, but after adding clinical variables (diabetes mellitus, hypertension, multivessel disease, SII, and eGFR), the AUC of the models increased to 0.84 and 0.83, respectively, suggesting that integrating multidimensional risk factors significantly improves the ability to identify lesion progression.^[11]^ In exploratory subgroup analyses, numerically higher effect estimates for both non-HDL-C and remnant-C were observed among patients with diabetes mellitus, multivessel disease, and impaired renal function. However, all interaction *P* values were >.05, and therefore there was no statistically significant evidence that the associations differed across these subgroups. These numerical differences should consequently be regarded as hypothesis-generating and require confirmation in adequately powered studies specifically designed to evaluate effect modification.

The predictive value of non-HDL-C and remnant-C for non-culprit lesion progression revealed in this study has a solid pathophysiological basis. Non-HDL-C represents the TC burden of all atherogenic lipoproteins, including LDL, very low-density lipoprotein, intermediate-density lipoprotein, and lipoprotein(a), all of which can enter the subendothelial layer of the arterial wall, be taken up by macrophages to form foam cells, and initiate and drive the formation and development of atherosclerotic plaques.^[12]^ Compared with LDL-C, non-HDL-C more comprehensively reflects the overall burden of atherogenic lipoproteins, with superior predictive advantages, particularly in the setting of elevated triglyceride levels or low LDL-C levels.^[13]^ Remnant cholesterol represents the cholesterol content of triglyceride-rich lipoproteins and their remnants; these particles are smaller in diameter, more atherogenic, and can directly penetrate the arterial endothelium and induce local inflammatory responses.^[14]^ Previous large-scale prospective studies have confirmed that elevated remnant-C levels are significantly associated with increased risks of coronary artery disease, ischemic stroke, and all-cause mortality, independent of LDL-C levels.^[15]^ The findings of this study further extend this chain of evidence to the field of functionally significant non-culprit lesion progression, suggesting that even among high-risk lesions with QFR ≤ 0.80, remnant-C remains a key lipid parameter determining prognosis.

Of note, patients with diabetes mellitus, multivessel disease, and renal dysfunction in this study exhibited a higher risk of lipid-related lesion progression. Patients with diabetes mellitus often present with an atherogenic lipoprotein profile, including increased small dense LDL particles, impaired clearance of triglyceride-rich lipoproteins, and impaired HDL function, and these metabolic disturbances collectively exacerbate the risk of non-culprit lesion progression.^[16]^ Multivessel disease reflects the severity of systemic atherosclerotic burden, and such patients often have a more active inflammatory state and a faster rate of plaque progression.^[17]^ Patients with renal dysfunction experience accelerated atherosclerosis due to the accumulation of uremic toxins, enhanced oxidative stress, and chronic microinflammation.^[18]^ The subgroup analysis results of this study suggest that for the above high-risk populations, stricter lipid management and more intensive imaging follow-up may yield greater clinical benefits. However, the present subgroup analyses are exploratory and do not establish subgroup-specific treatment effects or justify differential clinical management.

Regarding inflammatory and immune parameters, this study found that the SII was an independent predictor of lesion progression. SII, derived from platelet, neutrophil, and lymphocyte counts, is a novel, simple, and easily obtainable systemic inflammatory marker. Neutrophils and platelets play key roles in the initiation and progression of atherosclerotic plaques, while lymphocytes are involved in immune modulation.^[19]^ Previous studies have shown that elevated SII is significantly associated with coronary plaque vulnerability, in-stent restenosis, and adverse cardiovascular outcomes.^[20]^ This study further confirms that even among patients undergoing PCI and receiving statin therapy, residual inflammatory risk remains an important driver of non-culprit lesion progression, suggesting that combining anti-inflammatory strategies with lipid-lowering therapy may be a potential direction to further reduce residual risk.

The present study also found that baseline renal dysfunction (decreased eGFR) was an independent predictor of non-culprit lesion progression. There is a complex, bidirectional causal relationship between chronic kidney disease and atherosclerosis. Patients with renal dysfunction often have lipoprotein metabolism disorders, abnormal calcium-phosphorus metabolism, enhanced oxidative stress, and chronic inflammation, which collectively promote vascular calcification and plaque instability. Previous studies have shown that even mild to moderate renal dysfunction is associated with a significantly increased risk of cardiovascular events.^[21]^ The findings of this study suggest that renal function assessment should be a routine component of risk stratification after PCI, and more aggressive secondary prevention measures should be considered for patients with reduced eGFR.

The present study has the following limitations. First, this was a single-center retrospective study with a relatively limited sample size (n = 214), which may introduce selection bias; the conclusions need to be validated by larger-scale, multicenter prospective studies. In addition, only 62 patients experienced the primary outcome at the patient level. Given the number of covariates included in the multivariable models, the relatively limited number of outcome events may have resulted in overfitting and imprecision of the regression coefficients. Therefore, the multivariable findings should be regarded as exploratory and require validation in larger independent cohorts. Second, follow-up coronary angiography was not performed at standardized time points in all patients; some were clinically driven, which may introduce bias in the estimation of lesion progression rates. Third, remnant-C was estimated using a formula rather than directly measured; although this formula is widely accepted in clinical research, its accuracy is limited in patients with extremely high triglyceride levels (≥4.5 mmol/L); we plan to exclude such patients in sensitivity analyses to verify the robustness of the results. Fourth, the intensity of statins and other lipid-lowering medications may have changed during follow-up; although adjustments were made in the statistical analyses, the potential impact of medication adherence and dose adjustments on the results cannot be completely excluded. Fifth, this study did not assess plaque morphological features of non-culprit lesions (e.g., thin-cap fibroatheroma and plaque burden), and the association of these features with lesion progression requires further imaging studies.

In conclusion, non-HDL-C and remnant-C are independent predictors of progression of non-culprit coronary lesions with QFR ≤ 0.80. Combining these parameters with traditional risk factors significantly improves predictive performance for lesion progression. The findings of this study suggest that in patients with functionally significant non-culprit lesions after PCI, in addition to conventional LDL-C targets, attention should also be paid to non-HDL-C and remnant-C levels, which should serve as important reference indicators for risk stratification and individualized lipid-lowering therapy. Exploratory subgroup analyses showed numerically higher effect estimates in some clinically high-risk subgroups, but no statistically significant interactions were demonstrated. Further adequately powered studies are required to determine whether the associations of these lipid parameters differ according to diabetes status, extent of coronary disease, or renal function.

## Author contributions

**Conceptualization:** Guoran Ruan, Qianqian Guan, Jiaqing Li, Guo li, Haojin Ren.

**Data curation:** Guoran Ruan, Qianqian Guan, Jiaqing Li, Guo Li, Haojin Ren.

**Formal analysis:** Guoran Ruan, Qianqian Guan, Jiaqing Li, Guo Li, Haojin Ren.

**Funding acquisition:** Haojin Ren.

**Investigation:** Haojin Ren.

**Writing – original draft:** Haojin Ren.

**Writing – review & editing:** Haojin Ren.
